# Clinicopathological and Prognostic Implications of Epithelial‐to‐Mesenchymal Transition‐Related Immunohistochemical Markers in Resectable Pancreatic Cancer: A Retrospective Longitudinal Study

**DOI:** 10.1002/cnr2.70565

**Published:** 2026-05-03

**Authors:** Ahmad Machmouchi, Lama Zahreddine, Jessica Aoun, Noura Abbas, Maya Charafeddine, Laudy Chehade, Charbel Elias, Ziad El Husseini, Sally Temraz, Deborah Mukherji, Mohamad Khalife, Walid Faraj, Amar Zaidan, Nayrose Kadi, Ayman Tawil, Ali Shamseddine

**Affiliations:** ^1^ Department of Internal Medicine, Division of Hematology/Oncology, Naef K. Basile Cancer Institute American University of Beirut Beirut Lebanon; ^2^ Department of Pathology and Laboratory Medicine American University of Beirut Beirut Lebanon; ^3^ Department of Surgery American University of Beirut Beirut Lebanon

**Keywords:** E‐cadherin, EMT, immunohistochemistry, pancreatic cancer, pancreatic ductal adenocarcinoma, PDAC, vimentin

## Abstract

**Background:**

Pancreatic ductal adenocarcinoma (PDAC) is the sixth leading cause of global cancer death. The process of epithelial‐to‐mesenchymal transition (EMT) is a key driver of early progression and metastasis in PDAC.

**Aim:**

Our study aimed to explore the correlation between the expression of EMT markers and survival outcomes.

**Methods and Results:**

We conducted a retrospective longitudinal study on patients diagnosed with resectable PDAC between January 2005 and June 2019, with a 5‐year follow‐up for survival analysis. Immunohistochemical staining was performed to assess E‐cadherin and vimentin expression. EMT was defined as the presence of high Vimentin (mesenchymal) expression combined with low E‐cadherin (epithelial) expression. The study cohort included 135 patients with resectable PDAC, with 86 males (63.7%) and a mean age of 63.5 years (SD 10.1); most tumors were grade 2 (84, 64.6%). Cox regression analysis revealed that Vimentin expression (*p* = 0.005), positive margin (*p* = 0.008), and absence of metformin intake (*p* = 0.023) were independent predictors of poor OS. High Vimentin was associated with lower median OS (17.0 ± 4.4 vs. 25.8 ± 2.3 months, *p* = 0.037) and DFS (8.6 ± 1.2 vs. 13.0 ± 2.3 months, *p* = 0.014), compared to low Vimentin, and it was correlated with higher tumor grade (*p* = 0.028) and metastasis rate (*p* = 0.032). The poorest outcomes were observed when high Vimentin was coupled with low E‐cadherin (median OS of 12.6 ± 4.7 vs. 24.5 ± 2.1 months, *p* = 0.038; median DFS of 9.5 ± 0.5 vs. 10.8 ± 2.1 months, *p* = 0.029), compared to the rest of the population.

**Conclusion:**

Our findings showed that Vimentin overexpression and the EMT phenomenon are strongly associated with poor OS and DFS in resectable PDAC, underscoring their potential as prognostic biomarkers and therapeutic targets.

## 1 Introduction

Globally, pancreatic cancer ranks as the sixth leading cause of cancer‐related deaths, despite being the twelfth in incidence, as per the latest GLOBOCAN data [1]. The age‐standardized incidence and mortality rates of this disease are alarmingly close, both reaching a value of 3.9 per 100 000 in Lebanon, further highlighting its poor prognosis. Pancreatic ductal adenocarcinoma (PDAC) constitutes more than 90% of pancreatic cancer cases and remains one of the most lethal gastrointestinal malignancies [2]. Despite advances in treatment, survival rates have remained stagnant, underscoring the need for better prognostic markers [3].

Epithelial‐to‐mesenchymal transition (EMT), first identified during gastrulation, is a key process involved in cellular remodeling and tissue repair in normal tissues, but it can also play a pivotal role in driving tumor progression and metastasis in cancer cells [4, 5]. This transition has been observed in many tumors originating from epithelial cells, especially PDAC [4, 6]. EMT is a reversible process that allows immotile polarized epithelial cells to undergo biochemical changes, enabling them to acquire a mesenchymal cell phenotype [7, 8]. Through this process, EMT cells exhibit enhanced invasiveness, increased migratory capacity, resistance to apoptosis, and greater production of extracellular matrix components [8, 9]. EMT is regulated by molecular pathways, including transcription factors such as Snail, Slug, and ZEB1/2, which suppress epithelial markers like E‐cadherin (E‐cad), cytokeratin, and occludin, while enhancing mesenchymal markers such as Vimentin (Vim), N‐cadherin, and fibronectin [10]. Signaling pathways such as TGF‐β, Wnt/β‐catenin, PI3K/Akt, and hypoxia‐driven HIF‐1α stabilization further contribute to the invasiveness, metastatic potential, and resistance to apoptosis observed in PDAC cells. Notably, the downregulation of E‐cad coupled with the upregulation of Vim has been associated with poorer survival outcomes in pancreatic cancer patients [4].

E‐cad, a cell–cell adhesion molecule, is crucial in maintaining epithelial integrity and is a major suppressor of invasion and metastasis. Its downregulation during EMT disrupts cell–cell adhesion and contributes to tumor progression in pancreatic tumors [11]. Conversely, Vim, a type III filamentous protein predominantly expressed in mesenchymal cells, provides structural support and enhances cell integrity under stress [12]. Vim is recognized as a biomarker of mesenchymal transition and plays a key role in cellular processes such as migration, motility, adhesion, and resistance to multiple stressors. Its overexpression is commonly observed in metastatic pancreatic cancer [13, 14] and other malignancies, such as melanoma, lung, and breast cancers, where it has been associated with aggressive tumor behavior and poorer prognosis [15]. The functional switch from epithelial markers like E‐cad to mesenchymal markers like Vim is well‐established in malignancy and represents a hallmark of EMT [16]. Furthermore, Vim has been identified as a potential target for cancer therapy, though its therapeutic applications remain under investigation [17, 18].

Despite growing evidence, challenges remain in translating EMT biology into clinical practice due to variability in marker expression and uncertainties regarding its prognostic significance [19, 20]. We assessed the correlation between E‐cad and Vim expression, identified as EMT markers, and survival in patients with resectable PDAC who underwent surgery.

## 2 Materials and Methods

### 2.1 Patient Selection

This study was approved by the local Institutional Review Board (IRB) Committee at the American University of Beirut (AUB) (Approval number: BIO‐2029‐0300, Date: October 7, 2019) in accordance with ethical guidelines for biomedical research. A waiver of written consent was granted, and verbal consent was obtained from patients to perform immunohistochemical staining on archived tissue samples and to contact them for survival follow‐up. This procedure was approved by the IRB as the study involved no more than minimal risk, with no interventions outside the standard of care.

The study included patients aged 18 years and above, diagnosed with resectable PDAC, and who had undergone a Whipple, distal, or total pancreatectomy at the American University of Beirut Medical Center (AUBMC) between January 1, 2005, and June 30, 2019. All these patients had available formalin‐fixed, paraffin‐embedded blocks for immunohistochemical staining. Patients with neuroendocrine or other types of pancreatic cancer or presenting with unresectable PDAC were excluded from this study. Additionally, this work adheres to the Strengthening the Reporting of Observational Studies in Epidemiology (STROBE) guidelines (Table S1).

### 2.2 Methods

We collected clinical and pathological data, including patient demographics, comorbidities, and associated medications, tumor size, location, stage determined according to the AJCC TNM 8th edition, grade, margin status, lymphovascular invasion, treatment modalities, disease progression, metastatic sites, and survival status.

Immunohistochemical staining and analysis were performed on the formalin‐fixed, paraffin‐embedded tissue sections of 5 μm thickness, obtained from the blocks of resected pancreatic cancer, to document the presence of EMT. Deparaffinization and immunohistochemical staining were performed using a microwave streptavidin immunoperoxidase (MSIP) protocol and labeled streptavidin‐biotin (LSAB) method on a DAKO TechMate Horizon automated immunostainer. Monoclonal antibodies directed against E‐cad and Vim were validated for specificity through positive and negative controls and standardized protocols, ensuring reproducibility and consistency in immunohistochemical staining procedures. For Vim, tonsil tissue was used as the external positive control, demonstrating stromal and endothelial cell positivity with negative contrast in lymphocytes and epithelial lining. For E‐cad, normal epithelial elements within the tissue sections served as internal positive controls. Negative controls for both markers were performed by omitting the primary antibody. Scoring systems were rigorously cross‐validated to minimize interobserver variability. Pathology blocks used in the study were previously obtained as part of the standard clinical workup. The decision to focus on E‐cad and Vim as primary EMT markers was based on their well‐established roles in EMT biology, as well as considerations of feasibility and cost‐effectiveness.

The evaluation of marker expression included both the percentage of stained cells and staining intensity for E‐cad, and the percentage of stained cells for Vim. E‐cad staining intensity was scored as 0 (no stain), 1 (weak), 2 (moderate), or 3 (strong). The percentage of E‐cad‐positive cells was scored as 0 (< 5%), 1 (5%–25%), 2 (> 25%–50%), 3 (> 50%–75%), or 4 (> 75%). The E‐cad score was calculated by multiplying the percentage score by the intensity score, and expression was defined as low (1–3) or high (≥ 4), following a modified H‐score approach [21]. E‐cad immunostaining was specifically assessed at the cell membrane of tumor cells, consistent with its role as an epithelial adhesion molecule. Vim expression was categorized based solely on the percentage of stained cells, with low defined as < 10% and high as ≥ 10% [22, 23]. Vim immunostaining was assessed in the cytoplasm of tumor cells, reflecting its function as a mesenchymal intermediate filament protein. This scoring approach reflects the distinct biology of the markers: E‐cad often shows graded reduction and heterogeneous membranous staining, making the combined assessment of intensity and percentage more reliable; whereas Vim is de novo expressed with relatively uniform intensity once present, making the proportion of positive tumor cells the most reproducible and biologically relevant metric for EMT evaluation [17, 24].

EMT was defined as the presence of high Vim expression (mesenchymal marker) combined with low E‐cad expression (epithelial marker). Cases with both markers altered were classified as complete EMT, while those with only one marker altered were classified as partial EMT (Table S2).

### 2.3 Endpoints/Aims

In this study, we assessed the expression levels of E‐cad and Vim and examined their correlation with median overall survival (OS) and disease‐free survival (DFS) in patients with resectable PDAC. We also analyzed the clinicopathologic factors associated with survival outcomes and EMT marker expression to better understand their biological and clinical relevance.

The primary endpoint was to determine the correlation between EMT marker expression, particularly E‐cad and Vim, and median OS and DFS in patients with resectable PDAC. The secondary endpoints were to evaluate the association between EMT marker expression and clinicopathological characteristics, and to identify the patient and disease factors contributing to poor OS in this patient population.

We hypothesized that high Vim expression combined with low E‐cad expression would serve as predictors of poor outcome, both in terms of OS and DFS, and that EMT marker expression would be associated with more aggressive disease biology.

### 2.4 Follow‐Up and Survival

This retrospective study included a longitudinal component to assess survival outcomes over a 5‐year period. Survival data were obtained through hospital record review and direct contact with patients or family members via phone calls. Hospital records provided documentation of clinic visits, admissions, and disease progression, while phone calls were used to confirm survival status and obtain additional information when records were incomplete.

OS was defined as the time between the date of diagnosis and the date of death from any cause, or the last date of confirmed follow‐up for censored cases (who remained alive at last follow‐up). DFS was defined as the time from diagnosis to the first documented relapse, or to the last oncology follow‐up for censored cases (who remained disease‐free at last follow‐up).

### 2.5 Statistical Analysis

A priori sample size estimation was performed using a two‐sided log‐rank test with a significance level of 5% (*α* = 0.05) and 80% power (*β* = 0.20). Assuming a clinically relevant hazard ratio of approximately 1.5–2.0 between groups, a sample size of about 100 patients was required to detect statistically significant differences in survival outcomes.

OS and DFS curves were plotted using the Kaplan–Meier method, and log‐rank tests were used to assess differences between groups. Numerical variables were summarized by their median, mean, and range. Categorical variables were described by counts and relative frequencies.

Multivariable Cox regression analysis was performed to develop a prognostic model for survival, incorporating variables such as gender, age at diagnosis, E‐cad expression, Vim expression, low E‐cad with high Vim, neoadjuvant chemotherapy, staging at diagnosis, positive surgical margins, and metformin intake. Variables with substantial missing data, such as lymphovascular invasion (available for 92 patients only), were excluded to avoid weakening the model. Similarly, pT and pN were not separately included as they are captured within staging at diagnosis. Backward elimination was applied, with a removal threshold set at *p* > 0.1, to refine the model by eliminating the least significant variables iteratively until no further variables can be deleted without a statistically significant loss of fit. This approach ensured robust control of confounding factors while minimizing overfitting. Hazard ratios and 95% confidence intervals were calculated for variables that remained significant in the final model. Statistical analyses were conducted using the SPSS v.25.0 software package, with two‐sided *p* values and a significance threshold of *p* < 0.05.

## 3 Results

### 3.1 Demographic and Clinicopathological Characteristics

**FIGURE 1:**
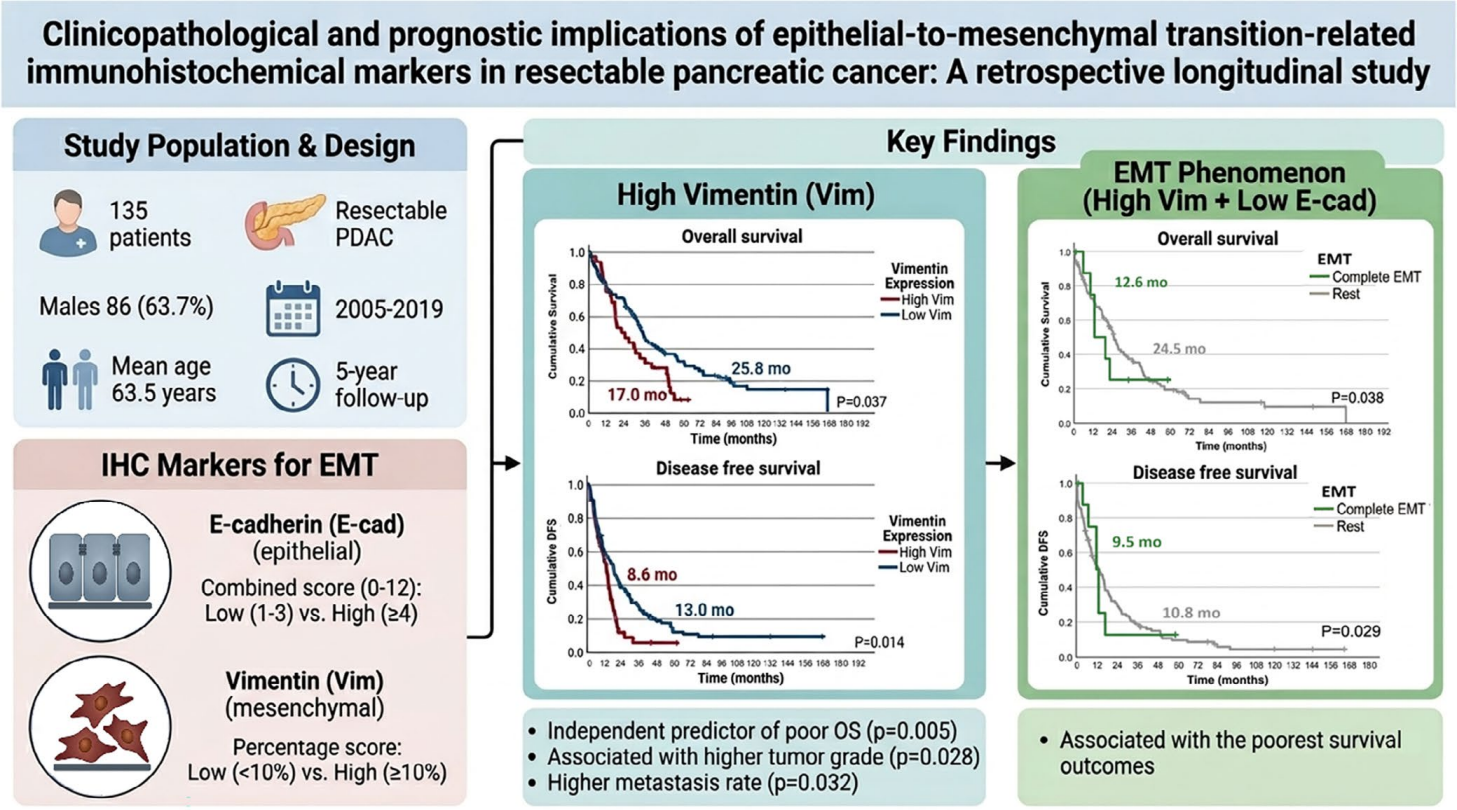
Visual summary of patient characteristics, study design, immunohistochemical assessment of EMT markers (E‐cadherin and Vimentin), and the prognostic implications of high Vimentin expression and the EMT phenomenon in resectable pancreatic ductal adenocarcinoma.

Between January 2005 and June 2019, 541 patients were diagnosed with pancreatic cancer at our center. Of these, 137 patients were identified as having resectable PDAC. Two cases were excluded due to the lack of paraffin blocks containing sufficient tumor tissue for immunohistochemical staining, resulting in 135 patients for the final analysis. The main findings from this study are illustrated in Figure 1.

The patient population comprised 49 females (36.3%) and 86 males (63.7%), with a mean age of 63.5 years (SD 10.1). The majority of patients were Lebanese (108, 80.0%), and the mean body mass index was 27.3 (SD 4.8). Tumor grade data were available for 130 patients: 19 patients (14.6%) had grade 1, 84 (64.6%) had grade 2, and 27 (20.8%) had grade 3. Most tumors were located at the head of the pancreas (104, 77.0%). Positive surgical margins were observed in 34 cases (25.6%). Stage information was available for 131 patients: 31 (23.7%) were diagnosed as stage I, 69 (52.6%) as stage II, and 31 (23.7%) as stage III. Seven patients (5.2%) received neoadjuvant treatment. Most patients underwent a Whipple procedure (116, 87.9%), while 16 (11.9%) underwent distal pancreatectomy and 3 (2.2%) underwent total pancreatectomy. Adjuvant chemotherapy was administered to 71/85 patients (83.5%), and 16/85 patients (18.8%) received radiotherapy (Table 1).

**TABLE 1:** Demographic and disease characteristics among 135 patients with resectable PDAC.

Characteristics	*N* (%) or mean (SD)
Demographics	
Age at diagnosis (years), mean (SD)	63.5 (10.1)
Gender, *N* (%)	
Male	86 (63.7)
Female	49 (36.3)
Nationality, *N* (%)	
Lebanon	108 (80.0)
Other	27 (20.0)
Body mass index, mean (SD)	27.3 (4.8)
Comorbidities and risk factors	
Smoker, *N* (%)	59 (53.6)
Waterpipe smoking, *N* (%)	5 (5.4)
Alcohol, *N* (%)	20 (18.0)
Hypertension, *N* (%)	62 (52.1)
Diabetes, *N* (%)	68 (50.4)
Metformin Yes	36 (52.9)
Metformin No	32 (47.1)
Hypertriglyceridemia, *N* (%)	34 (29.8)
Pancreatitis history, *N* (%)	28 (23.5)
Chronic kidney disease, *N* (%)	4 (3.5)
Heart disease, *N* (%)	21 (18.4)
Family history of pancreatic cancer, *N* (%)	1 (0.9)
Family history of other cancer types, *N* (%)	6 (5.5)
Tumor characteristics	
Tumor grade, *N* (%)	
Grade 1	19 (14.6)
Grade 2	84 (64.6)
Grade 3	27 (20.8)
Tumor location, *N* (%)	104 (77.0)
Head	8 (5.9)
Body	8 (5.9)
Tail	4 (3.0)
Uncinate process	2 (1.5)
Whole pancreas	1 (0.7)
Isthmus and head	1 (0.7)
Tail and body	1 (0.7)
Head and uncinate process	7 (5.2)
Tumor size (cm), mean (SD)	3.5 (1.4)
Positive margins, *N* (%)	34 (25.6)
Pathologic staging T, *N* (%)	
T1	18 (13.8)
T2	70 (53.4)
T3	35 (26.7)
T4	8 (6.1)
Pathologic staging N, *N* (%)	
N0	51 (38.0)
N1	60 (44.8)
N2	23 (17.2)
Staging at diagnosis, *N* (%)	
IA	11 (8.4)
IB	20 (15.3)
IIA	16 (12.2)
IIB	53 (40.4)
III	31 (23.7)
Lymphovascular involvement, *N* (%)	38 (41.3)
Treatment characteristics	
Neoadjuvant chemotherapy, *N* (%)	7 (5.2)
Operative procedure, *N* (%)	
Whipple procedure	116 (85.9)
Distal pancreatectomy	16 (11.9)
Total pancreatectomy	3 (2.2)
Adjuvant chemotherapy, *N* (%)	71 (83.5)
Radiotherapy, *N* (%)	16 (18.8)

### 3.2 EMT Marker Expression

In this cohort, most tumors demonstrated preserved E‐cad expression, with the majority showing moderate (61, 45.2%) or strong (47, 34.8%) staining intensity and a mean percentage of stained cells of 67.8% (SD 19.8). Overall, 107 patients (79.3%) had high E‐cad expression, while 28 (20.7%) had low expression. In contrast, Vim expression was less frequent, with a mean of 14.1% (SD 21.9) stained cells; 38 patients (28.1%) exhibited high Vim expression (≥ 10%), while 97 (71.9%) had low expression (< 10%) (Table 2).

**TABLE 2:** EMT marker expression among 135 patients with resectable PDAC.

EMT marker expression	*N* (%) or mean (SD)
E‐cadherin stain intensity (score), *N* (%)	
0 (No stain)	0 (0.0)
1 (Weak)	27 (20.0)
2 (Moderate)	61 (45.2)
3 (Strong)	47 (34.8)
E‐cadherin percentage of stained cells (%), mean (SD)	67.8 (19.8)
E‐cadherin percentage of stained cells (score), *N* (%)	
0 (< 5%)	0 (0.0)
1 (5%–25%)	5 (3.7)
2 (> 25%–50%)	19 (14.1)
3 (> 50%–75%)	51 (37.8)
4 (> 75%)	60 (44.4)
E‐cadherin score, mean (SD)	7.3 (3.5)
E‐cadherin expression, *N* (%)	
High (≥ 4)	107 (79.3)
Low (1–3)	28 (20.7)
Vimentin stained‐cell expression, mean (SD)	14.1 (21.9)
Vimentin expression, *N* (%)	
High (≥ 10%)	38 (28.1)
Low (< 10%)	97 (71.9)

When evaluating combinations of Vim and E‐cad expressions, the majority of patients had high E‐cad and low Vim (80, 59.3%) (no EMT), 27 patients (20.0%) displayed high expression for both markers (partial EMT), 17 patients (12.6%) had low expression for both markers (partial EMT), and 11 patients (8.1%) had a combination of high Vim with low E‐cad, which is characteristic of complete EMT (Table S2).

Cross‐tabulation analysis revealed that high Vim expression was significantly associated with higher tumor grade (*p* = 0.028) and metastasis rate (*p* = 0.032), as shown in Table 3. However, E‐cad expression did not show a significant correlation with prognostic characteristics.

**TABLE 3:** Clinicopathologic factors correlated with Vim and E‐cad expressions.

Characteristics	Vimentin (−), *n* (%)	Vimentin (+), *n* (%)	*p*	E‐cad low, *n* (%)	E‐cad high, *n* (%)	*p*
Age			0.251			0.527
< 65	44 (45.4)	22 (57.9)		12 (42.9)	55 (50.9)	
≥ 65	53 (54.6)	16 (42.1)		16 (57.1)	53 (49.1)	
Gender			0.843			1.000
Male	61 (62.9)	25 (65.8)		18 (64.3)	69 (63.9)	
Female	36 (37.1)	13 (34.2)		10 (35.7)	39 (36.1)	
Tumor grade			**0.028**			0.883
1	17 (18.3)	2 (5.40)		4 (15.4)	15 (14.4)	
2	60 (64.5)	24 (64.9)		16 (61.5)	68 (65.4)	
3	16 (17.2)	11 (29.7)		6 (23.1)	21 (20.2)	
Positive margins	25 (26.3)	9 (23.7)	0.829	6 (21.4)	28 (26.7)	0.635
Tumor stage			0.403			0.758
1	20 (21.5)	11 (28.9)		7 (25.0)	24 (23.3)	
2	50 (53.8)	19 (50.0)		15 (53.6)	54 (52.4)	
3	23 (24.7)	8 (21.1)		6 (21.4)	25 (24.3)	
Pathologic T stage			0.292			0.738
1	11 (11.8)	7 (18.4)		4 (14.3)	14 (13.5)	
2	39 (41.9)	18 (47.4)		12 (42.9)	46 (44.2)	
3	38 (40.9)	10 (26.3)		9 (32.1)	39 (37.5)	
4	5 (5.4)	3 (7.9)		3 (10.7)	5 (4.8)	
Lymphovascular invasion			0.244			0.292
No	36 (54.5)	18 (69.2)		8 (47.1)	46 (61.3)	
Yes	30 (45.5)	8 (30.8)		9 (52.9)	29 (38.9)	
Metastasis			**0.032**			0.299
No	29 (43.3)	5 (18.5)		5 (25)	30 (40)	
Yes	38 (56.7)	22 (81.5)		15 (75)	65 (60)	

### 3.3 Survival Analysis

Among 94 patients with available progression data, 70 (74.5%) experienced disease progression after surgery ± adjuvant treatment, with 60 (63.8%) developing distant metastasis. The most common metastatic sites were the liver (45/60, 75.0%), lung/pleura (19/60, 31.7%), and other sites (19/60, 31.7%), with some patients presenting multiple sites. Across the entire cohort, documented death during follow‐up was observed in 95 cases (70.4%).

At the 3‐year follow‐up, 34 patients (25.2%) were alive, 78 (57.8%) were deceased, and 23 (17.0%) were censored (alive at < 3 years). At the 5‐year follow‐up, 16 patients (11.9%) were alive, 89 (65.9%) were deceased, and 30 (22.2%) were censored (alive at < 5 years). According to Kaplan–Meier curves, the 3‐year and 5‐year survival percentages were 35.7% and 19.7%, respectively.

**FIGURE 2:**
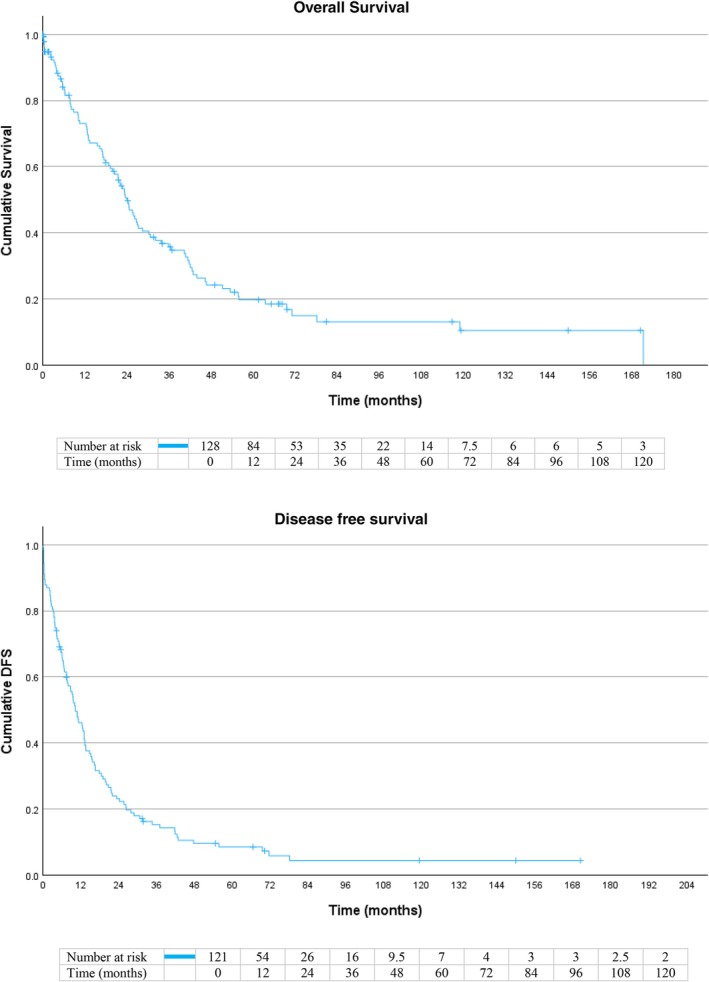
Kaplan–Meier curves for OS and DFS in the entire patient population with resectable PDAC.

**FIGURE 3:**
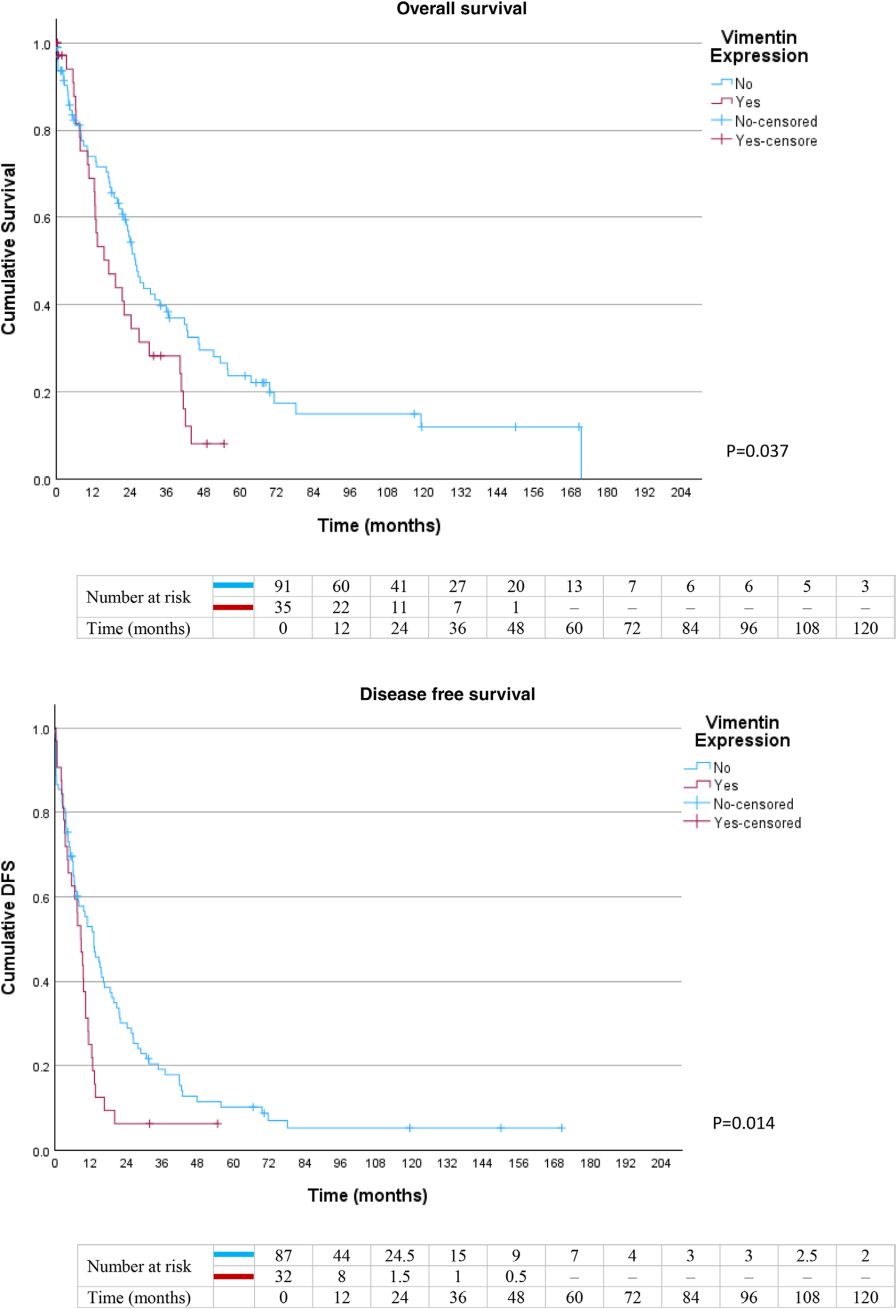
Kaplan–Meier curves for OS and DFS in patients with high versus low Vim expression.

The median OS for the entire patient population (135 patients) was 23.9 ± 2.0 months, while the DFS was 10.2 ± 1.5 months (Figure 2). High Vim expression was significantly associated with lower median OS (17.0 ± 4.4 vs. 25.8 ± 2.3 months, *p* = 0.037) and DFS (8.6 ± 1.3 vs. 13.0 ± 2.3 months, *p* = 0.014), compared to low Vim expression (Figure 3).

**FIGURE 4:**
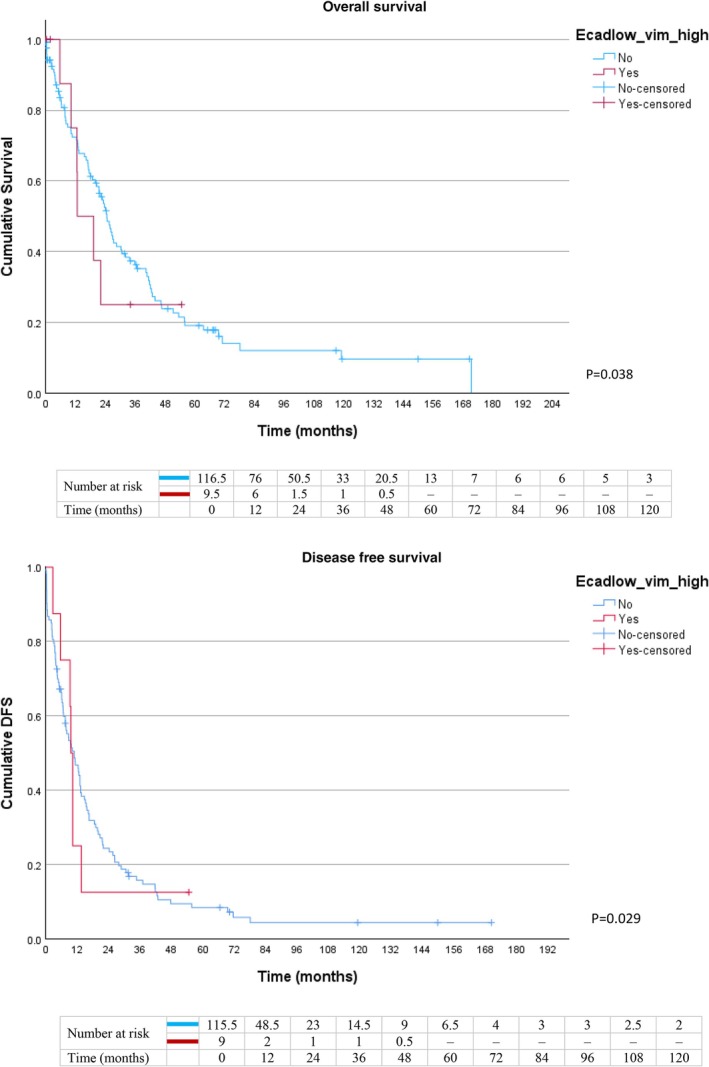
Kaplan–Meier curves for OS and DFS in patients with high Vim and low E‐cad versus rest of the population.

**FIGURE 5:**
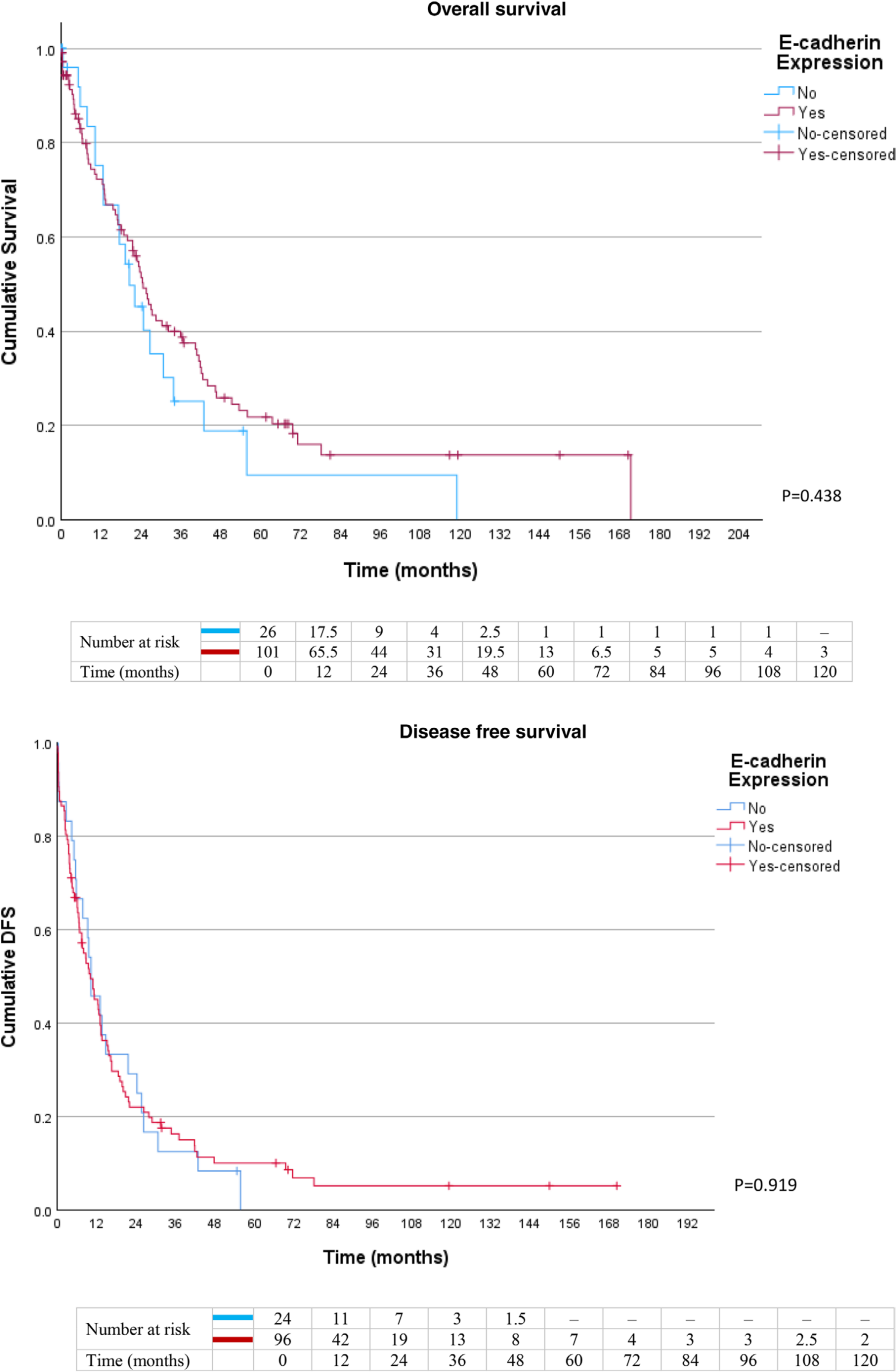
Kaplan–Meier curves for OS and DFS in patients with low versus high E‐cad expression.

Patients with high Vim and low E‐cad expression demonstrated the poorest survival outcomes, with lower median OS (12.6 ± 4.7 vs. 24.5 ± 2.1 months, *p* = 0.038) and DFS (9.5 ± 0.5 vs. 10.8 ± 2.1 months, *p* = 0.029), compared to the remaining patients with other marker profiles (Figure 4). Conversely, low E‐cad expression alone was associated with lower median OS (20.4 ± 4.1 vs. 24.3 ± 2.2 months) but did not achieve statistical significance (*p* = 0.438). Median DFS was comparable between low and high E‐cad expression groups (10.2 ± 2.3 vs. 10.0 ± 2.0 months, *p* = 0.919) (Figure 5).

In the multivariable Cox regression analysis, the variables that remained significant to the model were Vim expression, neoadjuvant chemotherapy, positive margin, staging, and the use of metformin (Table 4). High Vim expression (HR = 2.007, 95% CI = 1.234–3.263, *p* = 0.005), advanced stage (stage II) (HR = 1.860, 95% CI = 1.047–3.306, *p* = 0.034), positive margin (HR = 2.010, 95% CI = 1.196–3.378, *p* = 0.008), and absence of metformin intake (HR for metformin intake = 0.548, 95% CI = 0.326–0.919, *p* = 0.023) were statistically significant independent predictors of poor OS. Factors such as gender, age at diagnosis, E‐cad expression, and the specific combination of low E‐cad with high Vim were excluded through backward conditional elimination.

**TABLE 4:** Multivariable Cox regression analysis for overall survival (results of the final step in the backward conditional elimination process).

	Hazard ratio	95% CI		*p*
Vimentin expression	2.007	1.234	3.263	**0.005**
Neoadjuvant chemotherapy	0.214	0.029	1.562	0.129
Positive margin	2.010	1.196	3.378	**0.008**
Stage I				0.101
Stage II	1.860	1.047	3.306	**0.034**
Stage III	1.708	0.877	3.326	0.116
Metformin	0.548	0.326	0.919	**0.023**

*Note:* Variables entered in the initial model included gender, age at diagnosis, E‐cadherin expression, Vimentin expression, combination low E‐cadherin/high Vimentin, neoadjuvant chemotherapy, staging at diagnosis, positive surgical margins, and metformin intake. Variables with statistically significant *p*‑values in the final step of the backward conditional elimination process are shown in bold.

## 4 Discussion

This study highlights the prognostic significance of EMT markers, E‐cad and Vim, in patients with resectable PDAC, emphasizing their role in survival outcomes, tumor biology, and potential therapeutic implications. High Vim expression, a hallmark of mesenchymal phenotype, was strongly associated with poorer OS and DFS, while the combined high Vim and low E‐cad expression demonstrated significantly worse survival outcomes, reinforcing the prognostic relevance of EMT in PDAC.

Interestingly, our study found no significant association between low E‐cad expression alone and survival outcomes (Figure 5). This suggests that the loss of epithelial adhesion (partial EMT) may not be sufficient by itself to drive aggressive disease progression. In contrast, high Vim expression (Figure 3) was strongly associated with poor OS and DFS. The acquisition of mesenchymal traits, represented by Vim overexpression, acted as the dominant prognostic driver in PDAC. The most severe survival outcomes were observed in patients with both high Vim and low E‐cad expression (Figure 4), representing a complete EMT phenotype. Although the *p* values for this subgroup were comparable to those of Vim alone, these patients had the lowest median OS (12.6 months compared to 17.0 months in the overall high‐Vim group), indicating that combined epithelial loss and mesenchymal gain may synergistically accelerate tumor aggressiveness. The limited number of patients with this combined profile (*n* = 11) likely reduced statistical power, which may explain the lack of stronger statistical separation despite the biological plausibility.

Beyond binary EMT states, these findings emphasize the importance of considering a spectrum of states, including partial or hybrid EMT phenotypes. Emerging evidence shows that tumor cells can exist in intermediate states, retaining both epithelial and mesenchymal features, which may confer adaptability, metastatic potential, and therapy resistance. As noted in our study and supported by recent literature, complete EMT is rare; hybrid states (E+/M+), in which cells retain some E‐cad while gaining Vim, are particularly aggressive because they enable collective cell migration, where clusters of tumor cells move together rather than individually, resist apoptosis, and seed metastases more efficiently [25, 26]. This concept provides a mechanistic explanation for our Figure 4 findings and reinforces why high Vim remained an independent predictor of poor OS in multivariable analysis and was associated with higher tumor grade and metastasis rate in cross‐tabulation.

Moreover, EMT is not a permanent state but a dynamic, reversible process. Tumor plasticity allows tumor cells to dynamically shift between EMT and mesenchymal‐to‐epithelial transition (MET), adapting to therapeutic stress and microenvironmental cues [27]. The tumor microenvironment, including stromal interactions, hypoxia, and inflammatory signaling, further modulates EMT dynamics and may explain heterogeneity in clinical outcomes [28, 29]. Together, these insights highlight the complexity of EMT biology in PDAC and the need to interpret EMT markers within the broader context of tumor plasticity and microenvironmental influences.

Our findings align with prior studies that underscore the importance of EMT markers in cancer progression. For example, Wang et al. [30] demonstrated significantly worse OS and DFS for high Vim compared to low Vim among 120 patients with PDAC who received neoadjuvant therapy and underwent pancreatectomy. Similarly, Zhou et al. [31], while focusing on pancreatic neuroendocrine tumors, reported increased risks of distant metastasis, lymph node involvement, and perineural invasion among patients with high Vim and low E‐cad expression, further validating their prognostic significance. Wang et al. [32] additionally highlighted that interactions between Girdin and Vim could induce EMT, promoting tumor growth and metastasis in PDAC, further substantiating Vim's role as a marker of metastatic behavior. These results highlight the critical need for integrating EMT markers into standard risk stratification models to improve individualized patient management and guide treatment decisions in PDAC.

In our cohort, surgical specimens were used for EMT marker assessment rather than preoperative biopsies. This approach ensured consistency across patients, as not all had biopsy material available, and provided larger, more representative tissue samples for reliable immunohistochemical evaluation. While surgical specimens do not allow us to differentiate between patients who received treatment prior to surgery and those who were treatment‐naïve, the majority of patients (95%) underwent upfront surgery without prior therapy, which minimizes potential bias. The small proportion of patients who received neoadjuvant treatment (5%) likely affected its statistical significance and the reliability to assess its impact on EMT marker expression or survival outcomes. Accordingly, our findings primarily reflect the prognostic relevance of Vim expression in treatment‐naïve tumors.

Our multivariable Cox regression analysis identified Vim expression, positive surgical margins, and absence of metformin use as significant predictors of OS in PDAC. Specifically, patients with high Vim expression exhibited worse survival, consistent with Vim's established role in enhancing migration, invasion, and resistance to apoptosis [8, 9, 33]. Interestingly, our study showed that metformin use was associated with improved OS, suggesting a potential protective role, potentially counteracting EMT‐driven tumor aggressiveness. Preclinical studies by Gu et al. [34] demonstrated that metformin could reverse EMT through miR‐663 DNA methylation regulation, restoring epithelial characteristics and enhancing gemcitabine sensitivity in pancreatic cancer cells. However, randomized clinical trials evaluating metformin in PDAC have reported mixed results [35, 36, 37, 38], underscoring the need for further investigation into metformin's role as an adjunct treatment in PDAC.

In a previous study [39], we presented a unique case of pancreatic cancer with mesenchymal differentiation and positive Vim expression in a patient with primary lung adenocarcinoma. Despite achieving a complete response in lung disease, the patient died due to the progression of his pancreatic cancer, illustrating the aggressive nature of mesenchymal differentiation and its impact on survival. This case emphasizes the importance of incorporating molecular insights into clinical practice and integrating findings on EMT markers into actionable strategies for risk stratification and therapeutic intervention in PDAC.

This study has several limitations, including its retrospective design, the relatively small sample size, particularly for subgroups such as patients with low E‐cad/high Vim expression, reliance on immunohistochemistry for only two EMT markers (E‐cad and Vim), the absence of external validation, and limited molecular characterization of the cohort. Prospective studies with larger, molecularly profiled cohorts are needed to validate these findings and establish the clinical utility of EMT markers in PDAC prognosis. In addition, with the growing adoption of neoadjuvant therapy in resectable PDAC, particularly in patients with high‐risk features, future studies should specifically investigate how treatment influences EMT marker expression and its prognostic relevance, as our cohort primarily reflects treatment‐naïve tumors [40].

Future efforts should focus on elucidating the molecular mechanisms through which EMT drives tumor progression, therapeutic resistance, and metastasis. Improved understanding of these processes could pave the way for innovative treatments targeting the EMT pathway. Agents targeting Vim, such as monoclonal antibodies and small‐molecule inhibitors, are under investigation, though challenges related to drug delivery, toxicity, and marker variability remain significant barriers. Combining EMT‐targeted therapies with standard PDAC treatments could potentially improve therapeutic outcomes, and future clinical trials should explore the feasibility, safety, and efficacy of such approaches in this challenging disease context [41].

Our study contributes to the growing body of evidence supporting the relevance of EMT markers in PDAC and their potential impact on patient management. By integrating biomarkers such as Vim and E‐cad into prognostic models, clinicians can better identify high‐risk patients and tailor treatment strategies to improve outcomes. Furthermore, the exploration of EMT‐targeted therapies offers promising avenues for disrupting tumor progression and enhancing the efficacy of standard treatments.

## 5 Conclusion

Our study demonstrated that high Vim, a key EMT marker, was strongly associated with poor OS and DFS, as well as higher tumor grade and metastasis in PDAC patients who underwent resection. In addition, we identified other important factors associated with poor survival in PDAC, including advanced tumor stage at diagnosis, positive surgical margins, and absence of metformin intake. The poorest survival outcomes were observed when high Vim was combined with low E‐cad (complete EMT), highlighting the potential of EMT markers as prognostic biomarkers and therapeutic targets.

## Author Contributions

**Lama Zahreddine:** conceptualization, writing – original draft, writing – review and editing, methodology, investigation, visualization, formal analysis, data curation. **Ahmad Machmouchi:** conceptualization, methodology, data curation, writing – original draft, writing – review and editing, formal analysis, visualization, investigation. **Maya Charafeddine:** conceptualization, investigation, writing – original draft, writing – review and editing, visualization, methodology, formal analysis, data curation. **Laudy Chehade:** investigation, methodology, writing – review and editing, formal analysis, data curation, conceptualization. **Noura Abbas:** conceptualization, investigation, writing – original draft, writing – review and editing, visualization, methodology, formal analysis, data curation. **Amar Zaidan:** investigation, methodology, writing – review and editing, data curation. **Deborah Mukherji:** investigation, methodology, writing – review and editing, data curation. **Charbel Elias:** conceptualization, investigation, methodology, writing – review and editing, data curation. **Sally Temraz:** investigation, methodology, writing – review and editing, data curation. **Jessica Aoun:** conceptualization, investigation, writing – original draft, writing – review and editing, visualization, methodology, formal analysis, data curation. **Mohamad Khalife:** investigation, methodology, writing – review and editing, data curation. **Walid Faraj:** investigation, methodology, writing – review and editing, data curation. **Ali Shamseddine:** conceptualization, investigation, methodology, validation, writing – review and editing, formal analysis, project administration, supervision, data curation. **Nayrose Kadi:** investigation, methodology, writing – review and editing, data curation. **Ziad El Husseini:** conceptualization, investigation, methodology, writing – review and editing, data curation. **Ayman Tawil:** investigation, writing – review and editing, methodology, data curation.

## Funding

The authors have nothing to report.

## Disclosure

Part of this work was presented as an abstract and poster at the ESMO World Congress on Gastrointestinal Cancer 2023. Machmouchi A, Zahreddine L, Aoun J, Charafeddine M, Chehade L, Temraz S, Shamseddine A. P‐88 Epithelial‐to‐mesenchymal transition: An emerging prognostic tool in resectable pancreatic cancer, a retrospective study. Ann Oncol. 2023 Jun 1;34:S44–5.

## Ethics Statement

This study was approved by the Institutional Review Board at American University of Beirut (BIO‐2019‐0300, Date: October 7, 2019) and was conducted in accordance with the internationally accepted ethical standards. Verbal consent was obtained and recorded from all patients for record review, immunohistochemical staining, and follow‐up.

## Conflicts of Interest

The authors declare no conflicts of interest.

## Supporting information


**Table S1:** STROBE checklist for reporting observational studies.

**Table S2:** Distribution of Vim and E‐cad expressions among the 135 patients with resectable PDAC.

## Data Availability

The data that support the findings of this study are available from the corresponding author upon reasonable request.
