# Supplementary material for: Clinicopathological and Prognostic Implications of Epithelial‐to‐Mesenchymal Transition‐Related Immunohistochemical Markers in Resectable Pancreatic Cancer: A Retrospective Longitudinal Study
Source: Cancer Rep (Hoboken). 2026 May 3;9(5):e70565. doi: 10.1002/cnr2.70565 (PMC13135890; doi:10.1002/cnr2.70565)
Supplement: Supplementary file 1 — Table S1: STROBE checklist for reporting observational studies. [file CNR2-9-e70565-s001.docx]

STROBE Statement—checklist of items that should be included in reports of observational studies

|  | | | Item No. | Recommendation | Page  No. | | Relevant text from manuscript |
| --- | --- | --- | --- | --- | --- | --- | --- |
| **Title and abstract** | | | 1 | (*a*) Indicate the study’s design with a commonly used term in the title or the abstract | 1 | | Retrospective longitudinal study |
|  |  |  |  | (*b*) Provide in the abstract an informative and balanced summary of what was done and what was found | 2 | | Methods and Results sections in the Abstract |
| Introduction | | | | | | |  |
| Background/rationale | | | 2 | Explain the scientific background and rationale for the investigation being reported | 3-4 | | Section 1. Introduction |
| Objectives | | | 3 | State specific objectives, including any prespecified hypotheses | 4 | | Section 2. Specific aims |
| Methods | | | | | | |  |
| Study design | | | 4 | Present key elements of study design early in the paper | 5 | | Section 3.2. Methods and search strategy |
| Setting | | | 5 | Describe the setting, locations, and relevant dates, including periods of recruitment, exposure, follow-up, and data collection | 5-6 | | Section 3.2. Methods and search strategy  Section 3.3. Variables collected  Section 3.4. Follow-up and survival |
| Participants | | | 6 | (*a*) *Cohort study*—Give the eligibility criteria, and the sources and methods of selection of participants. Describe methods of follow-up  *Case-control study*—Give the eligibility criteria, and the sources and methods of case ascertainment and control selection. Give the rationale for the choice of cases and controls  *Cross-sectional study*—Give the eligibility criteria, and the sources and methods of selection of participants | 5-6 | | Section 3.1. Patient selection  Section 3.4. Follow-up and survival |
|  |  |  |  | (*b*) *Cohort study*—For matched studies, give matching criteria and number of exposed and unexposed  *Case-control study*—For matched studies, give matching criteria and the number of controls per case | 5 | | Section 3.1. Patient selection |
| Variables | | | 7 | Clearly define all outcomes, exposures, predictors, potential confounders, and effect modifiers. Give diagnostic criteria, if applicable | 5-6 | | Section 3.3. Variables collected  Section 3.5. Statistical analysis |
| Data sources/ measurement | | | 8* | For each variable of interest, give sources of data and details of methods of assessment (measurement). Describe comparability of assessment methods if there is more than one group | 5-6 | | Section 3.3. Variables collected  Section 3.5. Statistical analysis |
| Bias | | | 9 | Describe any efforts to address potential sources of bias | 6 | | Section 3.4. Follow-up and survival |
| Study size | | | 10 | Explain how the study size was arrived at | 6 | | Section 3.5. Statistical analysis |
| Quantitative variables | | 11 | | Explain how quantitative variables were handled in the analyses. If applicable, describe which groupings were chosen and why | 5 | | Section 3.3. Variables collected |
| Statistical methods | | 12 | | (*a*) Describe all statistical methods, including those used to control for confounding | 6 | | Section 3.5. Statistical analysis |
|  |  |  |  | (*b*) Describe any methods used to examine subgroups and interactions | 6 | | Section 3.5. Statistical analysis |
|  |  |  |  | (*c*) Explain how missing data were addressed | 6 | | Section 3.5. Statistical analysis |
|  |  |  |  | (*d*) *Cohort study*—If applicable, explain how loss to follow-up was addressed  *Case-control study*—If applicable, explain how matching of cases and controls was addressed  *Cross-sectional study*—If applicable, describe analytical methods taking account of sampling strategy | 6 | | Section 3.5. Statistical analysis |
|  |  |  |  | (*e*) Describe any sensitivity analyses | 6 | | Section 3.5. Statistical analysis |
| Results | | | | | | | |
| Participants | | 13* | | (a) Report numbers of individuals at each stage of study—eg numbers potentially eligible, examined for eligibility, confirmed eligible, included in the study, completing follow-up, and analysed | 7  15 | | Section 4. Results  Table 1 |
|  |  |  |  | (b) Give reasons for non-participation at each stage | 7 | | Section 4. Results |
|  |  |  |  | (c) Consider use of a flow diagram | 7  22 | | Section 4. Results  Supplementary Table S2 |
| Descriptive data | | 14* | | (a) Give characteristics of study participants (eg demographic, clinical, social) and information on exposures and potential confounders | 7  15 | | Section 4. Results  Table 1 |
|  |  |  |  | (b) Indicate number of participants with missing data for each variable of interest | 7  15 | | Section 4. Results  Table 1 |
|  |  |  |  | (c) *Cohort study*—Summarise follow-up time (eg, average and total amount) | 6 | | Section 3.4. Follow-up and survival (5 years follow-up) |
| Outcome data | | 15* | | *Cohort study*—Report numbers of outcome events or summary measures over time | 7  15 | | Section 4. Results  Table 1 |
|  |  |  |  | *Case-control study—*Report numbers in each exposure category, or summary measures of exposure | *Not applicable* | | |
|  |  |  |  | *Cross-sectional study—*Report numbers of outcome events or summary measures | *Not applicable* | | |
| Main results | | 16 | | (*a*) Give unadjusted estimates and, if applicable, confounder-adjusted estimates and their precision (eg, 95% confidence interval). Make clear which confounders were adjusted for and why they were included | 7  16 | | Section 4. Results  Table 2 |
|  |  |  |  | (*b*) Report category boundaries when continuous variables were categorized | 7  15 | | Section 4. Results  Table 1 |
|  |  |  |  | (*c*) If relevant, consider translating estimates of relative risk into absolute risk for a meaningful time period | *Not applicable* | | |
| Other analyses | 17 | | Report other analyses done—eg analyses of subgroups and interactions, and sensitivity analyses | | 7  17  In separate files | Section 4. Results  Table 3  Figures 1-4 | |
| Discussion | | | | | | | |
| Key results | 18 | | Summarise key results with reference to study objectives | | 8-9  10 | Section 5. Discussion  Section 6. Conclusion | |
| Limitations | 19 | | Discuss limitations of the study, taking into account sources of potential bias or imprecision. Discuss both direction and magnitude of any potential bias | | 9 | Section 5. Discussion | |
| Interpretation | 20 | | Give a cautious overall interpretation of results considering objectives, limitations, multiplicity of analyses, results from similar studies, and other relevant evidence | | 8-10 | Section 5. Discussion | |
| Generalisability | 21 | | Discuss the generalisability (external validity) of the study results | | 9-10 | Section 5. Discussion | |
| Other information | | |  | | | | |
| Funding | 22 | | Give the source of funding and the role of the funders for the present study and, if applicable, for the original study on which the present article is based | | *Not applicable* | | |

*Supplementary Table S1. STROBE checklist for reporting observational studies.*

*Give information separately for cases and controls in case-control studies and, if applicable, for exposed and unexposed groups in cohort and cross-sectional studies.

**Note:** An Explanation and Elaboration article discusses each checklist item and gives methodological background and published examples of transparent reporting. The STROBE checklist is best used in conjunction with this article (freely available on the Web sites of PLoS Medicine at http://www.plosmedicine.org/, Annals of Internal Medicine at http://www.annals.org/, and Epidemiology at http://www.epidem.com/). Information on the STROBE Initiative is available at www.strobe-statement.org.
