# Supplementary material for: Clinicopathological and Prognostic Implications of Epithelial‐to‐Mesenchymal Transition‐Related Immunohistochemical Markers in Resectable Pancreatic Cancer: A Retrospective Longitudinal Study
Source: Cancer Rep (Hoboken). 2026 May 3;9(5):e70565. doi: 10.1002/cnr2.70565 (PMC13135890; doi:10.1002/cnr2.70565)
Supplement: Supplementary file 2 — Table S2: Distribution of Vim and E‐cad expressions among the 135 patients with resectable PDAC. [file CNR2-9-e70565-s002.docx]

|  | **Low Vimentin, n (%)** | **High Vimentin, n (%)** | **Total, n (%)** |
| --- | --- | --- | --- |
| **Low E-cadherin, n (%)** | 17 (12.6) | 11 (8.1) | 28 (20.7) |
| **High E-cadherin, n (%)** | 80 (59.3) | 27 (20.0) | 107 (79.3) |
| **Total, n (%)** | 97 (71.9) | 38 (28.1) | 135 (100) |

Supplementary Table S2. Distribution of Vim and E-cad expressions among the 135 patients with resectable PDAC.

Complete EMT is characterized by high Vimentin with low E-cadherin, whereas partial EMT is characterized by either low or high expression of both markers.
